# Microalbuminuria and cardiorenal risk: old and new evidence in different populations

**DOI:** 10.12688/f1000research.17212.1

**Published:** 2019-09-19

**Authors:** Diego Francisco Márquez, Gema Ruiz-Hurtado, Julian Segura, Luis Ruilope

**Affiliations:** 1 Unidad de Hipertensión Arterial-Servicio de Clínica Médica, Hospital San Bernardo, Salta, Argentina; 2 Instituto de Investigación Imas12 and Unidad de Hipertensión, Hospital 12 de Octubre, Madrid, Spain; 3 Departamento de Medicina Preventiva y Salud Pública, Universidad Autónoma, Madrid, Spain; 4 Escuela de Estudios Postdoctorales and Investigación, Universidad de Europa de Madrid, Madrid, Spain

**Keywords:** albuminuria, cardiovascular risk, chronic kidney disease

## Abstract

Since the association of microalbuminuria (MAU) with cardiovascular (CV) risk was described, a huge number of reports have emerged. MAU is a specific integrated marker of CV risk and targets organ damage in patients with hypertension, chronic kidney disease (CKD), and diabetes and its recognition is important for identifying patients at a high or very high global CV risk. The gold standard for diagnosis is albumin measured in 24-hour urine collection (normal values of less than 30 mg/day, MAU of 30 to 300 mg/day, macroalbuminuria of more than 300 mg/day) or, more practically, the determination of urinary albumin-to-creatinine ratio in a urine morning sample (30 to 300 mg/g). MAU screening is mandatory in individuals at risk of developing or presenting elevated global CV risk. Evidence has shown that intensive treatment could turn MAU into normoalbuminuria. Intensive treatment with the administration of an angiotensin-converting enzyme inhibitor or an angiotensin receptor blocker, in combination with other anti-hypertensive drugs and drugs covering other aspects of CV risk, such as mineralocorticoid receptor antagonists, new anti-diabetic drugs, and statins, can diminish the risk accompanying albuminuria in hypertensive patients with or without CKD and diabetes.

## Introduction

Mogensen was the first to describe the importance of microalbuminuria (MAU) not only as a renal risk factor but also as a powerful predictor of cardiovascular (CV) mortality in patients with type 2 diabetes mellitus (T2DM) ^1^. Since the description by Mogensen, MAU has received special attention as a prognostic marker for CV or renal risk or both, even in non-diabetic subjects ^2^. Mounting evidence indicates a continuous relationship between urinary albumin excretion (UAE) and cardiorenal risk, similar to the relationship between blood pressure (BP) and risk of CV events ^3– 6^.

The presence of MAU implies dysfunction of the glomerular filtration barrier, which may result from hemodynamic-mediated mechanisms, functional or structural impairment of the glomerular barrier, or a combination of these ^7^. In normal conditions, UAE can change in day-to-day measurements. Moreover, age, sex, body mass index, a high-protein meal, and vigorous exercise can promote a transient increase in albumin excretion. On the other hand, pathological conditions such as fever, congestive heart failure, urinary tract infection, and some drugs can also increase albumin excretion, so we have to consider all of these confounding factors when analyzing the urinary excretion of albumin ^8^.

When MAU is present in pathological conditions, such as chronic kidney disease (CKD), diabetes, or hypertension (HTN), it represents the existence of renal target organ damage and is also a marker of CV morbidity and mortality and of progressive CKD. In this article, we will review the importance of MAU recognition and the management of this marker of cardiorenal risk.

## Microalbuminuria diagnosis

There are different types of analysis to assess the presence of MAU. The urine dipstick, an insensitive marker for albuminuria, does not become positive until albumin excretion exceeds 300 to 500 mg/day. In normal conditions, UAE is less than 30 mg/day. When this value oscillates between 30 and 300 mg/day in a 24-hour urine collection or 30 to 300 mg/g of creatinine (urine albumin-to-creatinine ratio, or UACR) in a first morning sample, we used the term MAU, also known as “moderately increased albuminuria”. When albuminuria is more than 300 mg/day, it is considered macroalbuminuria ^8, 9^. Although 24-hour urine collection is the gold standard for the detection of MAU, it has been suggested that screening can be carried out more simply. MAU can be tested from a first morning urine sample or at any time. In recent years, the albumin-to-creatinine ratio (ACR) from spot urine, preferably that first voided in the morning, may be considered equivalent to the values during a 24-hour urine collection ^7, 10– 12^.

## Microalbuminuria in different populations

### General “healthy” population

The prevalence of MAU in the general population varies from 2.2 to 11.8% in different studies ^13, 14^. In the Prevention of Renal and Vascular End-Stage Disease (PREVEND) study, a representation of the general population from Groningen (The Netherlands) participated in a survey through a postal questionnaire and a vial to collect an early-morning urine sample to determine MAU. A total of 40,856 subjects (47.8%) responded. MAU was present in 7.2% of the subjects, and after exclusion of the diabetic and hypertensive subjects, MAU was still prevalent in 6.6% of the subjects ^13^. Data from the US National Health and Nutrition Examination Survey (NHANES) showed an increase in prevalence of MAU (ACR of 30 to 300 mg/g) from 7.1 to 8.2% during the survey periods 1988 to 1994 and 1999 to 2004. The increase was attributed to older age of the population, prevalence of HTN and diabetes, and higher body mass index ^15^. MAU in a “healthy population” is not a benign condition, and the evidence has shown an association between MAU and increased CV risk and all-cause mortality ^16– 20^. In the above-mentioned PREVEND study ^13^, UAE is a predictor of all-cause mortality in the general population ^18, 19^. The Framingham Offspring Study of 1568 non-diabetic and non-hypertensive participants, which showed that low levels of UACR well below the current MAU threshold were associated with greater risk of CV disease (CVD), predicted the development of CVD (hazard ratio [HR] 1.36, 95% confidence interval [CI] 1 to 1.87) and of death (HR 1.55, 95% CI 1.10 to 2.20) ^5^.

Moreover, the presence of MAU was associated with increased risk of incident HTN and also diabetes ^21– 24^. There is strong evidence that the relationship between MAU and other CV risk factors, such as diabetes, HTN, left ventricular hypertrophy, hyperlipidemia, overweight, and metabolic syndrome, contributes to an increase in CV mortality ^6, 25^.

### Patients with diabetes

The classic definition of diabetic nephropathy is the presence of MAU that normally appears 5 to 10 years after the diagnosis of diabetes and that without adequate treatment progresses to end-stage renal disease (ESRD) ^26^. The cumulative incidences of MAU in patients with type 1 diabetes mellitus (T1DM) were 12.6% over 7.3 years, according to the European Diabetes (EURODIAB) Prospective Complications Study Group ^27^, and 33% in an 18-year follow-up study in Denmark ^28^. In patients with T2DM, the incidence of MAU showed an increase of 2.0% per year with a prevalence of 25% 10 years after diagnosis in the UK Prospective Diabetes Study (UKPDS) ^26, 29^. Based on UAE values, diabetic nephropathy has been categorized into stages: MAU and macroalbuminuria. Cumulative evidence suggests that the risk for developing diabetic nephropathy and CVD starts when UAE values are still within the normoalbuminuric range ^30– 32^. Although MAU has been considered a risk factor for macroalbuminuria, not all patients progress to this stage and some may regress to normoalbuminuria ^33, 34^. Moreover, recent evidence reported that almost 25% of patients with T2DM and CKD with little or no proteinuria have biopsy-proven diabetic nephropathy and can progress to ESRD. The cause of this change in profile of diabetic nephropathy is unclear ^35, 36^.

The natural history of diabetic nephropathy in patients with T1DM and T2DM is similar. However, the timing of diabetes diagnosis in T2DM is difficult to assess, and often target organ damage is present when the diagnosis of T2DM is confirmed. T1DM allows a timeline picture of kidney disease progression starting with MAU and proceeds through stages of overt proteinuria, kidney function decline, and end-stage kidney disease ^37, 38^. Nevertheless, this “classic” progression from MAU to macroalbuminuria is not seen in all patients. In fact, in patients with low levels of MAU (ACR of less than 100 mg/g) maintained for 2 to 3 years, it is possible to revert to normal UAE. Regression of MAU is facilitated by improved glucose, BP, and lipid control ^38, 39^. On the other hand, epidemiological evidence unequivocally shows that albuminuria is a marker of subclinical renal damage and is associated with the progression of CKD as well as CVD in patients with diabetes ^29, 32, 39– 41^. The relationship between albuminuria and decline of glomerular filtration rate (GFR) has been documented in many studies ^26^. In a 4-year study that included 194 Pima Indians with T2DM, the rate of GFR decreased by 3% in those with MAU at baseline ( *P* = 0.29) and by 35% in those with macroalbuminuria ( *P* <0.001) ^38^. Ninomiya *et al*. showed that T2DM patients with both a UACR of 300 mg/g and an estimated GFR (eGFR) of less than 60 mL/minute per 1.73 m ^2^ had a 3.2-fold higher risk for CV events and a 22.2-fold higher risk for renal events compared with patients with neither of these risk factors ^42^. These findings are similar in other ethnic patients with diabetes ^43^.

In an interesting study published recently, Minutolo *et al*. ^44^ compared the risk of all-cause mortality, fatal and non-fatal CV events, and ESRD between CKD patients with (n = 693) and without (n = 1481) diabetes. The authors stratified participants according to proteinuria level (<0.15, 0.15 to 0.49, 0.5 to 1, and >1 g/day). During 4 years of follow-up, in the subgroup with UAE of less than 0.15 g/day, the risks of ESRD, CV events, and mortality were similar in diabetic and non-diabetic patients. Conversely, in patients with diabetes and CKD, the mortality risk was higher in patients with albuminuria of 0.15 to 0.49 g/day (HR 1.92, 95% CI 1.25 to 2.95), 0.5 to 1 g/day (1.99, 95% CI 1.26 to 3.15), and more than 1 g/day (1.98, 95% CI 1.28 to 3.06) ^44^.

### Arterial hypertension

HTN is an established risk factor of CV and all-cause mortality ^45– 47^. The prevalence of MAU in arterial HTN varies in different studies, ranging from 8 to 15% ^48– 51^. However, in some studies, the prevalence was much higher ^52^ and this wide difference may be due to differences in urine collection, cutoff level of normal albumin excretion, analytical methods, and the presence of anti-hypertensive medications.

Many studies have shown an association between MAU and BP circadian profile ^53– 55^. Ambulatory BP monitoring has shown that nighttime BP levels are associated with MAU ^56^ and were a better predictor of major adverse CV events than office BP and 24-hour or daytime levels ^57, 58^. In the general population, sustained HTN was associated with albuminuria. Moreover, the Hisayama study showed the association of white coat HTN and masked HTN with albuminuria in the general Japanese population ^59^. Similar findings were found for masked HTN ^60, 61^. In resistant hypertensive patients, Oliveras *et al*. found that true resistant HTN was associated with silent target organ damage, especially albuminuria ^62^.

The European guidelines for HTN emphasize the importance of assessing the presence of organ damage for CV risk stratification, including the estimation of albuminuria ^63^. So it is very important that physicians look for the presence of MAU or subclinical renal involvement in patients with HTN to better assess CV risk stratification and develop strategies to control BP and reduced proteinuria ^64^.

### Target organ damage

Different authors found an association between MAU as a marker of subclinical organ damage in non-diabetic hypertensive patients. The presence of MAU in patients with HTN was associated with a higher prevalence of left ventricular geometric patterns, especially concentric hypertrophy, assessed by either electrocardiography or echocardiography, compared with normoalbuminurics ^65– 67^. The presence of MAU is also associated with several vascular structural and functional alterations ^68, 69^, such as higher thickness of the intima and media of carotid artery compared with patients with normoalbuminuria ^70^, and predicts the development and progression of carotid atherosclerosis ^71^.

MAU was also associated with retinal vascular damage in patients with HTN ^72^. In patients with T1DM, MAU is a powerful predictor for the development of proliferative diabetic retinopathy and blindness ^73^. MAU was also identified as a marker of coronariopathy in diabetic and non-diabetic patients and in the general population and as a marker of cardiac dysfunction ^74– 77^.

## Chronic kidney disease

CKD is recognized as a major global public health problem ^3, 78^. CKD affects 10 to 16% of the adult population and is an important risk factor for CVD events, particularly when albuminuria is present ^3^. Available clinical practice guidelines have emphasized the use of current level of albuminuria as well as eGFR for CKD definition and staging ^79, 80^. Different studies have shown the detrimental role that albuminuria plays in CKD progression and CV mortality in these populations ^81^. Sumida *et al*. showed, in a cohort of 56,946 US veterans with eGFR of more than 60 mL/minute per 1.73 m ^2^, that relative changes in albuminuria over a 1-year interval were linearly associated with subsequent risk of kidney outcomes ^82^. Similarly, in patients with diabetes, MAU and macroalbuminuria are associated with higher risks of CVD and of reduced eGFR compared with normoalbuminuria ^83^.

As the worsening of albuminuria leads to CKD progression, its reduction with specific treatment leads to improving kidney function, so that albuminuria could be considered a therapeutic target in clinical practice and a surrogate endpoint for ESRD ^84, 85^. In the Stockholm CREAtinine Measurements (SCREAM) project, 31,732 individuals were studied with two or more ambulatory ACR tests to assess changes between ACR, ESRD, or death. Compared with stable ACR, a fourfold increase in ACR was associated with a 3.08-fold (95% CI 2.59 to 3.67) higher risk of ESRD and mortality, whereas a fourfold decrease in ACR was associated with a 0.34-fold (0.26 to 0.45) lower risk of ESRD ^86^. Different authors have documented mortality and morbidity associated with albuminuria changes ^87, 88^.

In kidney transplant recipients, Weiner *et al*. recently reported that the presence of albuminuria (MAU and macroalbuminuria) in kidney transplant recipients was strongly associated with graft failure, CVD events, and death ^89^. This association is similar to that seen between albuminuria outcomes in the general population ^89^.

In recent decades, the BP target for hypertensive patients with CKD and proteinuria has been changing constantly. A few years ago, the recommended BP targets for the CKD population were less than 140/90 mm Hg and, if albuminuria was present, less than 130/80 mm Hg ^79, 90– 92^. The publication of the Systolic Blood Pressure Intervention Trial (SPRINT) in 2015 contributed to the consideration of a lower BP goal in non-diabetic CKD ^93^. SPRINT is a landmark study that randomly assigned 9361 persons without diabetes to a systolic BP (SBP) target of less than 120 mm Hg (intensive treatment) versus an SBP of less than 140 mm Hg (standard treatment). The authors concluded that targeting an SBP of less than 120 mm Hg, compared with less than 140 mm Hg, resulted in lower rates of fatal and non-fatal major CV events and death from any cause ^93^. In that trial, 28% of the study population had CKD (eGFR of 20 to less than 60 mL/minute per 1.73 m ^2^), and in that group, intensive BP management seemed to provide the same benefits for reduction in the CVD composite primary outcome and all-cause mortality as were seen in the full study cohort ^93^. Given that most patients with CKD die from CVD complications, SPRINT evidence supports a lower target of less than 130/80 mm Hg for all patients with CKD ^93, 94^. However, observational studies of CKD cohorts indicate a higher risk of mortality at lower systolic pressures in elderly patients with CKD ^95, 96^. In contrast, in the pre-specified subgroup analysis of the elderly cohort in SPRINT, frail elderly patients did sustain benefit from the lower BP target, which supports a lower goal for all patients, including those with CKD ^97^. In contrast, data from the Action to Control Cardiovascular Risk in Diabetes (ACCORD) study indicate that strict BP control (SBP of less than 120 mm Hg) did not improve renal outcomes in patients with T2DM ^98^. Since the publication of SPRINT, different guidelines have reviewed the BP target in the CKD population and support the BP target of less than 130/80 mm Hg for CKD patients independently of the presence of albuminuria ^63, 99^.

## Management of albuminuria: focus on pharmacotherapy

MAU is frequently present in asymptomatic patients. Recently, two meta-analyses assessed albuminuria as a surrogate endpoint for CKD progression in randomized controlled trials ^100, 101^. Both articles found that the range in albuminuria was consistently associated with risk of end-stage kidney disease, lending support to the use of change in albuminuria as a surrogate endpoint for end-stage kidney disease ^100, 101^. Given that MAU as a marker of target organ damage is associated with CV mortality and CKD progression, it is important to treat these patients intensively.

## Renin–angiotensin–aldosterone system blockade intervention trials

Reduction of albuminuria under anti-hypertensive treatment is associated with reduced risk of clinical CV events and at the same time renal protection ^102, 103^. There is cumulative evidence of the efficacy of angiotensin-converting enzyme inhibitors (ACEis) and angiotensin receptor blockers (ARBs) for the treatment of HTN in this population ^63, 99, 104, 105^. A few trials that focus the intervention on MAU have been designed. In the Action in Diabetes and Vascular Disease (ADVANCE) trial, perindopril/indapamide was effective in preventing the onset of MAU, progression of MAU to macroalbuminuria, and even regression of albuminuria compared with placebo in patients with T2DM ^106^. Similarly, in the Randomized Olmesartan and Diabetes Microalbuminuria Prevention (ROADMAP) trial, olmesartan diminished the onset of MAU by 23% in T2DM ^107^. Dual renin–angiotensin–aldosterone system (RAAS) blockade between an ACEi plus an ARB should be avoided ^63, 99^. This combination showed more cases of acute kidney injury and hyperkalemia without benefits ^108, 109^. A similar result was found with the addition of the direct renin inhibitor aliskiren to an ACEi or ARB ^110^. The combination of the standard therapy with an ACEi or an ARB with a mineralocorticoid receptor antagonist (MRA) is an interesting option for managing patients with albuminuria but has the inconvenience of frequent hyperkalemia in patients with CKD ^111, 112^. In order to avoid the limitations of hyperkalemia of the traditional RAAS blockers in patients with CKD, new drugs have been developed ^113^. The new non-steroidal MRA finerenone (BAY948862) has better MRA selectivity than spironolactone and also reduced albuminuria and end organ damage more effectively than eplerenone ^114^. In the MinerAlocorticoid Receptor Antagonist Tolerability Study (ARTS), finerenone reduced albuminuria from baseline levels and had a lower incidence of hyperkalemia when compared with spironolactone in patients with chronic heart failure ^115^. Moreover, in the MinerAlocorticoid Receptor Antagonist Tolerability Study-Diabetic Nephropathy (ARTS-DN) study, different oral doses of finerenone in patients with T2DM reduced albuminuria ^116^. The addition of the new potassium binder patiromer ^117^ and the selective cation exchanger sodium zirconium cyclosilicate ^118^ facilitated the use of traditional MRA in patients with CKD, reducing potassium levels ^119^.

## Glucose-lowering agents

In recent years, new anti-diabetic agents appeared not only showing benefits in glycemic control but also reducing CV mortality and improving kidney function. The SGLT2 inhibitor acts as a glycosuric agent. In the Empagliflozin, Cardiovascular Outcomes, and Mortality in Type 2 Diabetes trial (EMPA-REG OUTCOME), empagliflozin or placebo was added to standard care in patients with T2DM and CVD ^120^. Empagliflozin reduced new or worsening nephropathy, doubling of serum creatinine (1.5% of the empagliflozin versus 2.6% placebo; *P* <0.001%), and progression to macroalbuminuria (11.2% empagliflozin versus 16.2% placebo; *P* <0.001%) ^121^. Similarly, in the CANagliflozin cardioVascular Assessment Study (CANVAS), canagliflozin or placebo was added to standard care in patients with T2DM with CVD or at high risk of CVD ^122^. Active treatment with canagliflozin slowed the rate of eGFR decline and reduced UACR. Doubling of serum creatinine and new-onset macroalbuminuria were significantly reduced ^122^.

Moreover, in the recently published Canagliflozin on Renal and Cardiovascular Outcomes in Participants With Diabetic Nephropathy study (CREDENCE), patients with T2DM and CKD were randomly assigned to receive canagliflozin or placebo ^123^. The study was ended earlier because of recommendations from the safety committee. After 2.62 years, 4401 patients were randomly assigned. The event rates in the canagliflozin group and the placebo group were 43.2 and 61.2 per 1000 patient-years, respectively (HR 0.70, 95% CI 0.59 to 0.82; *P* = 0.00001), and the relative risk of the primary outcome was 30% lower in the canagliflozin group. The relative risk of end-stage kidney disease was lower by 32% (HR 0.68, 95% CI 0.54 to 0.86; *P* = 0.002), and the relative risks of the renal-specific composite of end-stage kidney disease, a doubling of the creatinine level, or death from renal causes were lower by 34% (HR 0.66, 95% CI 0.53 to 0.81; *P* <0.001). The canagliflozin group had lower risks of hospitalization for heart failure (HR 0.61, 95% CI 0.47 to 0.80; *P* <0.001) and CV death, myocardial infarction, or stroke (HR 0.80, 95% CI 0.67 to 0.95; *P* = 0.01) ^123^.

Two multicenter phase III trials with SGLT2 inhibitor looking for renal and CV endpoints are ongoing: the EMPA-Kidney with empaglifozin (ClinicalTrials.gov Identifier: NCT03594110) and the Dapa-CKD with canagliflozin (ClinicalTrials.gov Identifier: NCT03036150). Both studies aim to investigate the effect of SGLT2 inhibitors on kidney disease progression or CV death versus placebo.

GLP-1 agonists liraglutide ^124^ and semaglutide ^125^ have also shown a significant improvement in CV outcomes of patients with T2DM and were accompanied by a more significant decrease in body weight and a less important drop in BP in the absence of natriuretic effects. This last point could explain the absence of effects on heart failure. Liraglutide also exhibited a renal protective capacity through a significant decrease in albuminuria ^126^.

## Endothelin receptor antagonists

Endothelin receptor antagonists reduce albuminuria and BP but can also cause sodium retention. A previous trial with the non-selective endothelin receptor antagonist avosentan in patients with diabetes and CKD was stopped prematurely because of an increased incidence of heart failure ^127^. By contrast, short-term treatment with low doses of the more selective endothelin A receptor antagonist atrasentan reduced albuminuria without causing significant fluid retention ^128, 129^. Recently, the Study of Diabetic Nephropathy with Atrasentan (SONAR) was published and showed a reduction in the risk of renal events with no significant differences for hospitalization for heart failure and also mortality compared with placebo ^130^.

## Statins and fibrates

Drugs other than anti-hypertensives can influence MAU and renal function. Different meta-analyses showed reductions of MAU, proteinuria, and clinical deaths with statins ^131– 133^. Fibrates showed also a reduction in albuminuria ^134^.

## Conclusions

MAU is a specific integrated marker of CV risk and target organ damage in patients with HTN, CKD, and diabetes and its recognition is important for identifying patients at higher risk for CV mortality. The determination of albumin in 24-hour urine collection is the gold standard, but screening is usually made with the UACR (30 to 300 mg/g) in the first voiding urine. Using ACEi or ARB in these patients is recommended and recently more strategies for its management, such as the new anti-diabetic drugs or the novel MRA, have become available. At the moment, diagnosis of MAU is challenging because of the possibility of returning to normoalbuminuria with intensive treatment.
